# CatLet score and clinical CatLet score as predictors of long-term outcomes in patients with acute myocardial infarction presenting later than 12 hours from symptom onset

**DOI:** 10.1080/07853890.2024.2349190

**Published:** 2024-05-13

**Authors:** Yong-Ming He, Shinichiro Masuda, Ting-Bo Jiang, Jian-Ping Xu, Bei-Chen Sun, Jun-Bo Ge

**Affiliations:** a Division of Cardiology, The First Affiliated Hospital of Soochow University, Jiangsu, China; b Department of Cardiology, National University of Ireland, Galway, Ireland; c Division of Cardiology, Zhongshan Hospital, Fudan University, Shanghai, China

**Keywords:** Angiographic scoring, clinical variables, coronary artery disease, prognosis

## Abstract

**Background:**

Our recently developed ***C***oronary ***A***rtery ***T***ree description and ***L***esion ***E***valua***T***ion (CatLet) angiographic scoring system is unique in its description of the variability in the coronary anatomy, the degree of stenosis of a diseased coronary artery, and its subtended myocardial territory, and can be utilized to predict clinical outcomes for patients with acute myocardial infarction (AMI) presenting ≤12 h after symptom onset. The current study aimed to assess whether the Clinical CatLet score (CCS), as compared with CatLet score (CS), better predicted clinical outcomes for AMI patients presenting >12 h after symptom onset.

**Methods:**

CS was calculated in 1018 consecutive AMI patients enrolled in a retrospective registry. CCS was calculated by multiplying CS by the ACEF I score (age, creatinine, and left ventricular ejection fraction). Primary endpoint was major adverse cardiac events (MACEs) at 4-year-follow-up, a composite of cardiac death, myocardial infarction, and ischemia-driven revascularization.

**Results:**

Over a 4-year follow-up period, both scores were independent predictors of clinical outcomes after adjustment for a broad spectrum of risk factors. Areas-under-the-curve (AUCs) for CS and CCS were 0.72(0.68–0.75) and 0.75(0.71–0.78) for MACEs; 0.68(0.63–0.73) and 0.78(0.74–0.83) for all-cause death; 0.73(0.68–0.79) and 0.83(0.79–0.88) for cardiac death; and 0.69(0.64–0.73) and 0.75(0.7–0.79) for myocardial infarction; and 0.66(0.61–0.7) and 0.63(0.58–0.68) for revascularization, respectively. CCS performed better than CS in terms of the above-mentioned outcome predictions, as confirmed by the net reclassification and integrated discrimination indices.

**Conclusions:**

CCS was better than CS to be able to risk-stratify long-term outcomes in AMI patients presenting >12 h after symptom onset. These findings have indicated that both anatomic and clinical variables should be considered in decision-making on management of patients with AMI presenting later.

## Introduction

For the evaluation of a coronary lesion, at least two key aspects should be included: the degree of the lesion and the myocardial territory subtended by the diseased coronary artery. Many methods have been introduced and intensively studied to accurately quantify the degree of stenosis and its associations with clinical outcomes [1]. However, very few studies have examined the subtended blood supply territory of a diseased coronary artery solely based on coronary angiography (CAG). In 1981, Leaman et al. developed a coronary scoring system based on the severity of luminal diameter narrowing and weighted it according to the usual flow to the left ventricle in each coronary artery [2]. This early pioneering work, considering both the stenosis degree and the involved myocardial territory, ultimately formed the basis of the Synergy between Percutaneous Coronary Intervention with Taxus and Cardiac Surgery (SYNTAX) score [3]. Although a large application of the SYNTAX score has been achieved with respect to risk stratification and optimal revascularization strategies for patients with coronary artery disease [4–6], there are some conflicting reports on the prognostic values of this anatomic score [7–9]. Refinement of the anatomic score with the subtended myocardial territory could possibly be achieved. To the best of our knowledge, our recently developed ***C***oronary ***A***rtery ***T***ree description and ***L***esion ***E***valua***T***ion (CatLet) angiographic scoring system, available at www.catletscore.com, is the first anatomic scoring system that has adequately accommodated the variability in the coronary anatomy and considers both the stenosis of a diseased coronary artery and its subtended myocardial territory [10]. Our preliminary study revealed that the CatLet score (CS), with satisfactory inter- or intra-observer reproducibility, better predicted the clinical outcomes for patients with acute myocardial infarction (AMI) than the anatomical SYNTAX score, although this novel scoring tool has not been tested in the context of randomized trials [11–13]. The current study hypothesized that the Clinical CatLet score (CCS), as compared with CS, had a better predictive value in AMI patients presenting later than 12 h after symptom onset.

## Methods

### Patients

Consecutive patients presenting later than 12 h with chest discomfort, accompanied by an elevated cardiac troponin I level (>99% upper limit normal), with a diagnosis of suspected acute myocardial infarction (ST or non-ST-segment elevation), admitted from January 1, 2012, to September 30, 2015, were retrospectively enrolled and underwent a CAG examination. AMI was diagnosed based on the third universal definition of myocardial infarction [14]. Patients presenting with at least one lesion with ≥50% diameter stenosis in vessels >1.5 mm in diameter were included. The percutaneous coronary intervention (PCI) procedure was performed using standard techniques for all included patients. In this institution, immediate PCI (<2 h) is usually performed for patients at very high risk. Patients at high risk usually undergo PCI within 24 h while those at intermediate or low risk usually undergo PCI within 72 h [15]. In our hospital, culprit lesions are usually approached in the index procedure while non-culprit lesions are usually approached in the staged procedure. The median time of hospitalization were eight days. Drug-eluting stents were used in the present study. Maintenance therapy included lifelong aspirin (100 mg/d) and clopidogrel (75 mg/d) for at least one year unless contraindicated. The exclusion criteria were as follows: Valvular heart disease, coronary artery bypass graft surgery, coronary embolism, normal CAG or coronary diameter stenosis <50%, prior stenting, chronic total occlusion, not receiving CAG, repeat hospitalizations, non-Chinese, coronary anomaly, prior myocardial infarction, thyroid dysfunction, poor CAG images, and lost to follow up.

This study complied with the Declaration of Helsinki regarding investigation in humans and was approved by the Institutional Review Board of Soochow University. The Institutional Review Board waived the need for the written informed consent before analysis because of its retrospective nature. This extended validation study for the CatLet angiographic scoring system was registered at chictr.org.cn (ChiCTR2000033730).

### CS and CCS

The CatLet angiographic scoring system has been described in detail elsewhere [10]. In short, this comprehensive angiographic scoring system was developed based on the 17 myocardial segmental model, the law of competitive blood supply, and the law of flow conservation, and attempted to account for the anatomic diversity of the coronary artery trees and to semi-quantify the myocardial territory subtended by the diseased coronary arteries [10,16]. According to the CatLet angiographic scoring system, 54 types of coronary circulation patterns were used to cover the diversity of the coronary anatomy, and the coronary segments were weighted according to the coronary circulation pattern and the law of flow conservation. Three variables of left descending artery, diagonals, and right coronary artery will define a specific coronary circulation pattern for an individual. The weightings of coronary segments will automatically display for further evaluation of the lesions using the online calculator. A typical case for the score calculation has been illustrated in Figure S1–S4 (Supplementary Materials Online). The lesion score is a product of the weighting factor of the coronary segment and its degree of stenosis (2.0 for non-occlusive lesions and 5.0 for occlusive lesions). The scores for separate lesions were then added to derive the total score. The CatLet angiographic scoring system worked completely according to the results of CAG, and had three characteristics [10]: (1) to reflect the variability in the coronary anatomy; (2) to consider the stenosis of a coronary artery and its subtended myocardial territory simultaneously; and (3) only a stenosis lesion was scored, while those adverse angiographic characteristics pertinent to the lesion, such as thrombus burden, lesion length, angulation, and bifurcation lesion, were no longer scored, but rather qualitatively recorded. These adverse angiographic characteristics were reported to not be consistently associated with clinical outcomes, except for heavy calcification and bifurcation lesions, and hence deserving of being separately investigated [17–19].

Two interventional cardiologists calculated CS. In case of disagreement, a third opinion was sought, and a final decision was made by consensus (*Kappa* = 0.86) [12]. Investigators calculating CS were blinded to the patients’ baseline data and clinical outcomes. Non-occlusive lesions were scored upfront; for a total occlusive lesion, wiring/small ballooning was used to improve the blood flow sufficiently to evaluate the severity of the lesion, and additionally possible diseases downstream; persistently poor blood flow that failed to allow adequate visualization of the lesion was scored as a total occlusive lesion [20]. In the current study, the scores for the non-occlusive status were analyzed. The ACEF I (age, creatinine, and left ventricular ejection fraction (LEVEF) score was computed as follows: age (years)/LVEF (%) +1 (serum creatinine level ≥2 mg/dL) [21]. CCS was calculated by multiplying CS by the ACEF I score [6].

### Outcome assessment

The primary endpoint was a composite of major adverse cardiac events (MACEs), including cardiac death, myocardial infarction, and ischaemia-driven revascularization for target or non-target lesions, and the secondary endpoints were all-cause death, cardiac death, myocardial infarction, and ischaemia-driven revascularization. The investigators collected source documents of all endpoint events for adjudication by an independent events committee. All patients were followed up until the date of these endpoints or at four year, whichever came first. Patients with adverse events were usually hospitalized , and their medical records, discharge summaries, and angiographic data were thus systematically reviewed for verifying these events. For deceased patients, death information was obtained from the household registration management system, hospitals, or the next of kin; if conflicting death information existed, the cause of death provided by the hospital was used. All undetermined deaths were cardiac, unless a non-cardiac cause was established clinically or at autopsy. Ischemia-driven revascularization was defined as diameter stenosis ≥50% in the presence of ischemic signs or symptoms or ≥70%, even in the absence of ischemic signs or symptoms. For patients with multiple laboratory examinations during hospitalization, the lowest value for creatinine and the highest value for cardiac troponin I were collected. LVEF, measured using transthoracic echocardiography during hospitalization, usually on the second day of admission, was used for the analysis.

### Statistical analysis

Statistical analyses were performed using STATA/SE 15 (State Corp LP, College Station, TX, USA). Multivariate imputation using chained equations (25 times) imputes missing entries based on the associations among and distributions of variables in this dataset [22]. Continuous variables were presented as mean ± standard deviation (SD) or median values (25^th^-75^th^ percentile), as appropriate; categorical variables were presented as frequencies and percentages. Comparisons were performed using the Kruskal–Wallis rank test or Pearson’s chi-square test, as appropriate. Testing for trends in event rates across tertiles was completed using the STATA procedure Opartchi. Event-free survival curves were generated using the Kaplan–Meier method, and survival rates among the tertiles were compared using the Log-rank test. Cox regression analysis was performed to identify the associations between the predictors and clinical outcomes. The model performance was characterized by discrimination and calibration. Receiver operating characteristic (ROC) curves were used to compare the discrimination between CS and CCS. The predicted performance of CS or CCS was internally validated using 10-fold cross-validation [23]. The agreement between the observed and predicted risks was assessed visually using calibration plots and quantitatively using the Hosmer–Lemeshow test. We added a lowess smooth curve to each calibration plot based on a locally weighted regression of the observed frequency on the expected probability. A cutoff equal to the event rate is used for binary classification, and cutoffs equal to half the event rate, the event rate, and twice the event rate were used when more than two categories were desired [24]. In the current study, the MACEs rate was 23.0% (234/1018). Therefore, half the event, the event, and twice the event rates were 10%, 20% and 40%, respectively. These three cutoffs will be used to categorize the patients in this study: ≤10%, 10% to ≤20%, 20% to ≤40% and >40%. Using this risk category, the category-dependent net reclassification improvement (NRI) was calculated. The category-free NRI, independent of the arbitrary choice of categories, deemed any changes in predicted risk in the correct direction as appropriate. We also calculated the integrated discrimination improvement (IDI), which integrated the NRI over all possible cutoffs of predicted risk and mathematically corresponded to the difference in discrimination slopes of the two models for comparison [25]. All tests were two-sided, and a *P*-value <0.05 was considered statistically significant.

## Results

**Figure 1.**
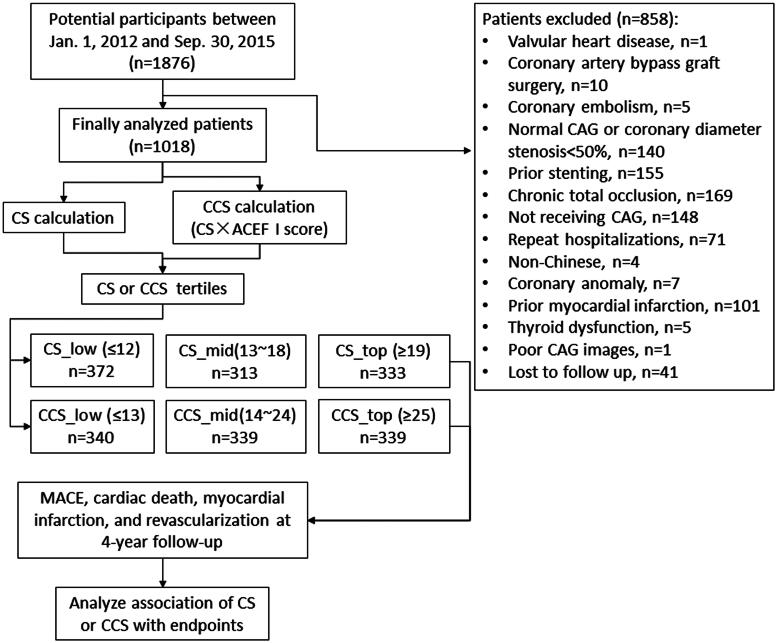
Flowchart of patient enrolment. CS = CatLet score; CCS = Clinical CatLet score; MACE = major adverse cardiac events; and CAG = coronary angiography.

A total of 1920 consecutive patients were initially identified for potential analysis, among whom 858 patients were excluded, and 1018 patients met the inclusion criteria as shown in Figure 1. There were 234 MACEs, 130 all-cause deaths, 93 cardiac deaths, 133 myocardial infarctions, and 131 ischemia-driven revascularizations at four years of follow-up. The 1018 patients had a total of 1730 lesions, with an average of 1.70 ± 0.94 lesions/patient. CS ranged from 2 to 47.5, with a mean ± SD of 16.38 ± 8.10, and a median (IQR) of 14 (10-21). CCS ranged from 2 to 136, with a mean ± SD of 22.36 ± 15.65 and a median (IQR) of 17.73(11.37-29.11). Both scores were non-parametric, and their distributions were right-skewed. The tertiles of CS and CCS were as follows: CS_low ≤12, CS_mid 13–18, CS_top ≥19, CCS_low ≤13, CCS_mid 14–24, and CCS_top ≥25, respectively. Baseline characteristics and angiographic data stratified according to the presence or absence of MACEs were shown in Table 1.

**Table 1.** Baseline clinical characteristics and angiographic data.

Factors	Missing	MACE-free	MACE	P-value
N		784	234	
Age (years), median (IQR)		65.00 (56.00, 72.00)	71.00 (63.00, 78.00)	<0.01
Male sex, *n*(%)		635 (80.99)	167 (71.37)	<0.01
ST-segment elevation MI, *n*(%)		385 (49.11)	123 (52.56)	0.35
Body mass index, median (IQR)	13.56%	23.88 (21.97, 26.09)	24.22 (22.20, 26.26)	0.52
Primary Hypertension, *n*(%)		504 (64.29)	169 (72.22)	0.02
Type 2 diabetes mellitus, *n*(%)		166 (21.17)	70 (29.91)	<0.01
Prior stroke, *n*(%)		40 (5.10)	21 (8.97)	0.03
Smoking, *n*(%)				<0.01
Never		270 (34.44)	113 (48.29)	
Past		72 (9.18)	29 (12.39)	
Current		442 (56.38)	92 (39.32)	
Alcohol intake, *n*(%)				<0.01
Never		561 (71.56)	190 (81.20)	
Past		27 (3.44)	9 (3.85)	
Current		196 (25.00)	35 (14.96)	
ST-segment elevation MI, *n*(%)		385 (49.11)	123 (52.56)	0.35
LDL-C, mmol/L, median (IQR)	1.28%	2.49 (2.10, 3.09)	2.39 (1.99, 3.20)	0.26
Troponin I, ng/mL, median (IQR)	0.59%	6.35 (1.64, 21.60)	7.52 (1.79, 35.40)	0.11
Creatinine, umol/L, median (IQR)	0.69%	71.00 (61.00, 83.00)	74.00 (61.70, 92.00)	0.02
Ejection fraction, %, median (IQR)	5.30%	54.00 (46.00, 63.00)	48.00 (42.00, 58.00)	<0.01
Dx, n(%)				0.05
Inter.		566 (72.19)	154 (65.81)	
Large		143 (18.24)	60 (25.64)	
Small		75 (9.57)	20 (8.55)	
LAD, *n*(%)				0.16
Average		460 (58.67)	140 (59.83)	
Long		230 (29.34)	57 (24.36)	
Short		94 (11.99)	37 (15.81)	
Dominance, n(%)				0.11
Average RCA		264 (33.67)	72 (30.77)	
Large RCA		197 (25.13)	59 (25.21)	
PDA only		45 (5.74)	19 (8.12)	
PDA zero		41 (5.23)	11 (4.70)	
Small RCA		203 (25.89)	53 (22.65)	
Super RCA		34 (4.34)	20 (8.55)	
Heavy calcification, *n*(%)		68 (8.67)	49 (20.94)	<0.01
No. of lesions, median (IQR)		1.00 (1.00, 2.00)	2.00 (1.00, 3.00)	<0.01
Culprit LAD, *n*(%)		420 (53.57)	133 (56.84)	0.38
Culprit LCX, *n*(%)		150 (19.13)	49 (20.94)	0.54
Culprit RCA, n(%)		223 (28.44)	71 (30.34)	0.57
Culprit LM, *n*(%)		20 (2.55)	11 (4.70)	0.09
Lesion length > 20mm, *n*(%)		101 (12.88)	54 (23.08)	<0.01
Aortal ostial lesion, *n*(%)		23 (2.93)	24 (10.26)	<0.01
Severe torturosity, *n*(%)		335 (42.73)	116 (49.57)	0.06
High thrombus burden, *n*(%)		423 (53.95)	131 (55.98)	0.58
Angulation < 70, *n*(%)		210 (26.79)	93 (39.74)	<0.01
Trifurcation lesion, *n*(%)		3 (0.38)	0 (0.00)	0.34
Bifurcation lesion, *n*(%)		346 (44.13)	135 (57.69)	<0.01
LAD, *n*(%)		424 (54.08)	120 (51.28)	0.45
LCX, *n*(%)		154 (19.64)	42 (17.95)	0.56
RCA, *n*(%)		252 (32.14)	80 (34.19)	0.56
LM, *n*(%)		20 (2.55)	9 (3.85)	0.30
1-vessel disease, *n*(%)		498 (63.52)	88 (37.61)	<0.01
2-vessel disease, *n*(%)		217 (27.68)	88 (37.61)	<0.01
3-vessel disease, *n*(%)		62 (7.91)	54 (23.08)	<0.01
LM disease, *n*(%)		29 (3.70)	21 (8.97)	<0.01
Length of stents, median (IQR)		23.00 (15.00, 30.00)	20.00 (0.00, 30.00)	0.28
No. of stents, median (IQR)		1.00 (1.00, 1.00)	1.00 (0.00, 1.00)	0.28
Complete revascularization		510 (65.05)	84 (35.90)	<0.01

Notes: CatLet= Coronary Artery Tree description and Lesion Evaluation and Treatment System, BMI = body mass index; SBP = systolic blood pressure; LDL-C = low density lipoprotein-cholesterol, Dx = diagonals, LAD = left anterior descending artery, RCA = right coronary artery, LCX = left circumflex, LM = left main, and PDA = posterior descending artery.

### Clinical outcomes, CS or CCS, and their associations

As CS had a close relationship with CCS, each score was entered separately in the multivariable analysis. There were no collinearity issues among the potential predictors. Age, serum creatinine, and left ventricular ejection fraction, being components of ACEF, and hence of CCS, were excluded out of the models to minimize collinearity.

On a continuous scale, CS was associated with increased four-year hazard ratios (95%CI)/SD of 1.90(1.70-2.12) for MACE, 1.90(1.64-2.20) for all-cause death, 2.17(1.83-2.57) for cardiac death, 1.88(1.63-2.18) for myocardial infarction, and 1.70(1.46-1.98) for ischemia-driven revascularization; Equivalents for CCS were 1.98(1.81-2.16), 2.22(2.00-2.47), 2.36(2.11-2.64), 2.07(1.86-2.31), and 1.55(1.34-1.80). After adjustment for a broad spectrum of risk factors, these associations were attenuated, but remained significant (*P* all values <0.001), as shown in Table 2 and Table S1 (Supplementary Materials Online).

**Table 2.** Hazard ratios (95%CI) for CatLet score or Clinical CatLet score with respect to clinical outcomes on a categorical or continuous scale.

						Crude HR(95%CI)					Adjusted HR(95%CI)			
	Continuous scale	Categorical scale				Continuous scale	Categorical scale							
Outcomes	*Per* SD CS higher	CS_low (≤12)	CS_mid (13 ∼ 18)	CS_top (≥19)	*P* for trend	*Per* SD CS higher	CS_low (≤12)	CS_mid (13 ∼ 18)	CS_top (≥19)	*P* for trend
MACE	1.90(1.70–2.12)	1.00	1.74(1.16–2.61)	4.82(3.39–6.85)	<0.01	1.70(1.51–1.92)	1.00	1.54(1.02–2.31)	3.80(2.64–5.49)	<0.01
All-cause death	1.90(1.64–2.20)	1.00	1.59(0.94–2.69)	4.04(2.56–6.36)	<0.01	1.53(1.31–1.80)	1.00	1.18(0.69–2.02)	2.56(1.59–4.13)	<0.01
Cardiac death	2.17(1.83–2.57)	1.00	1.80(0.89–3.64)	6.17(3.39–11.24)	<0.01	1.75(1.46–2.10)	1.00	1.28(0.62–2.63)	3.95(2.11–7.38)	<0.01
Myocardial infarction	1.88(1.63–2.18)	1.00	1.70(1.01–2.88)	4.32(2.73–6.84)	<0.01	1.66(1.42–1.95)	1.00	1.44(0.85–2.46)	3.35(2.08–5.40)	<0.01
Revascularization	1.70(1.46–1.98)	1.00	1.75(1.05–2.93)	4.12(2.61–6.50)	<0.01	1.66(1.41–1.95)	1.00	1.72(1.03–2.88)	3.87(2.40–6.24)	<0.01
	*Per* SD CCS higher	CCS_low (≤13)	CCS_mid (14 ∼ 24)	CCS_top (≥25)	*P* for trend	*Per* SD CCS higher	CCS_low (≤13)	CCS_mid (14 ∼ 24)	CCS_top (≥25)	*P* for trend
MACE	1.98(1.81–2.16)	1.00	1.91(1.24–2.93)	5.82(3.96–8.54)	<0.01	1.93(1.75–2.13)	1.00	1.78(1.15–2.74)	5.03(3.36–7.51)	<0.01
All-cause death	2.22(2.00–2.47)	1.00	2.18(1.15–4.13)	7.97(4.53–14.02)	<0.01	2.13(1.90–2.40)	1.00	1.89(1.00–3.60)	5.82(3.22–10.51)	<0.01
Cardiac death	2.36(2.11–2.64)	1.00	2.59(1.01–6.68)	14.55(6.32–33.48)	<0.01	2.36(2.08–2.67)	1.00	2.33(0.90–6.03)	11.72(4.98–27.58)	<0.01
Myocardial infarction	2.07(1.86–2.31)	1.00	1.57(0.86–2.85)	6.16(3.71–10.24)	<0.01	2.07(1.84–2.33)	1.00	1.50(0.82–2.73)	5.67(3.33–9.63)	<0.01
Revascularization	1.55(1.34–1.80)	1.00	1.70(1.03–2.80)	3.56(2.24–5.64)	<0.01	1.48(1.25–1.75)	1.00	1.62(0.98–2.68)	3.23(1.98–5.26)	<0.01

For CatLet score, adjusted for age, sex, primary hypertension, type 2 diabetes mellitus, prior stroke, smoking, serum creatinine, left ventricular ejection fraction, and heavy calcification; for Clinical CatLet score, adjusted for similar risk factors except that age, serum creatinine, and left ventricular ejection fraction were left out of the final models as the CCS already included these three clinical risk factors. MACE = major adverse cardiac events, SD = standard deviation, CS = CatLet score, and CCS = Clinical CatLet score. 1 SD is 8.1 for CatLet score and 15.6 for Clinical CatLet score, respectively.

**Figure 2.**
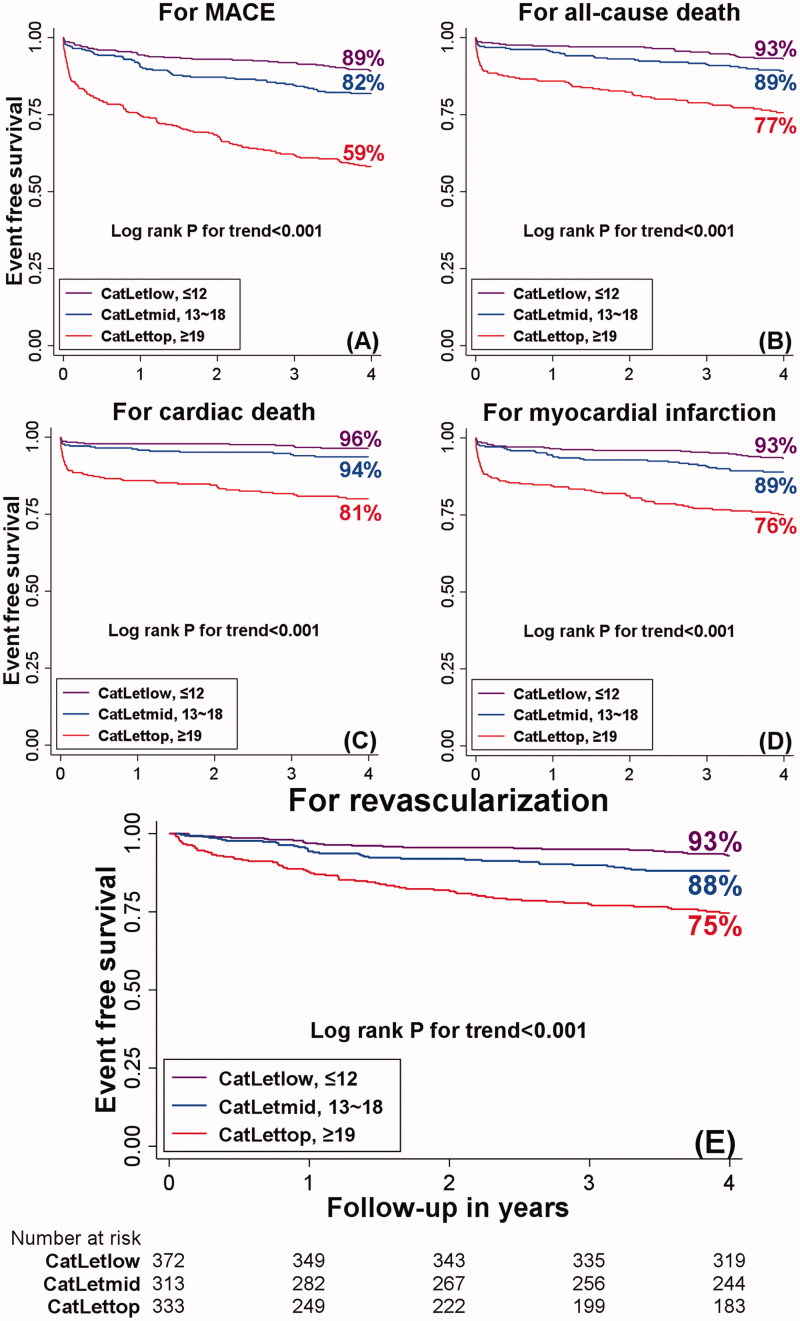
Kaplan–Meier curves for MACE (A), all-cause death (B), cardiac death (C), myocardial infarction (D), and revascularization (E) at four years according to the CatLet score tertiles.

On a categorical scale, four-year hazard ratios (95%CI) for MACE were significantly higher with CS_top than with CS_low (4.82(3.39-6.85), ***p* **<** **0.001), which was also the case for all-cause death, cardiac death, myocardial infarction, and ischemia-driven revascularization; for all these endpoints, there was a significant trend (*p* < 0.001) for higher event rates with increasing CS tertiles as shown in Figure 2(A–E). Stratifying outcomes across CCS tertiles resulted in similar results for the comparisons between CCS_top, CCS_mid, and CCS_low. Both scores remained significantly associated with an increased risk of all endpoints with increasing tertiles after adjustment for a broad spectrum of risk factors (Table 2).

### CS vs. CCS

**Figure 3.**
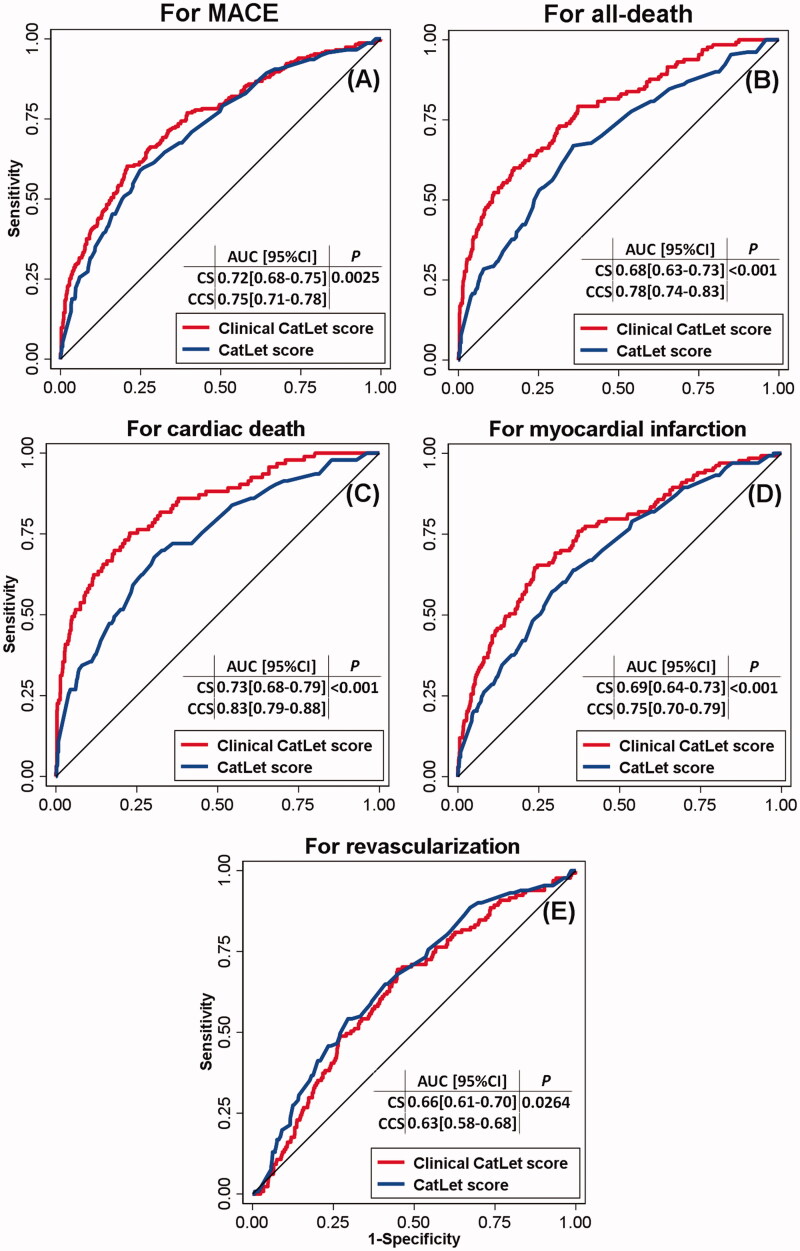
ROC curves for Clinical CatLet score and CatLet score, respectively, with respect to MACE (A), all-cause death (B), cardiac death (C), myocardial infarction (D), and revascularization (E). MACE = major adverse cardiac events; AUC = area-under-the-curve; CS = CatLet score; CCS = Clinical CatLet score.

The ROC curves for MACE, all-cause death, cardiac death, myocardial infarction, and ischemia-driven revascularization were shown in Figure 3(A–E). For MACE, CCS, as compared with CS, had a significantly increased Areas-under-the-curve (AUCs) (0.75[0.71–0.78] vs. 0.72[0.68–0.75], *p* = 0.0025), which was also the case for all-cause death, cardiac death, and myocardial infarction. However, for ischemia-driven revascularization, CCS had a significantly decreased AUC (0.63[0.58–0.68] vs. 0.66[0.61–0.7], *p* = 0.0264).

**Figure 4.**
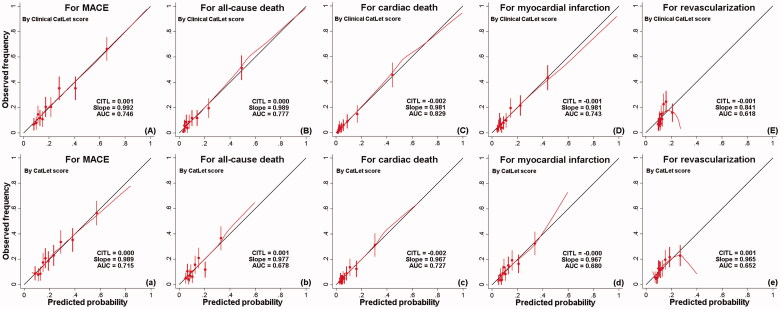
Calibration plots at cross-validation for Clinical CatLet score and CatLet score, respectively, with respect to MACE (A, a), all-cause death (B, b), cardiac death (C, c), myocardial infarction (D, d), and revascularization (E, e). Intercept was also called CITL (calibration-in-the-large). A lowess smoothing curve was added to each calibration plot. Intercept of 0 and slope of 1 indicated a perfect prediction. Negative and positive intercepts indicated overestimation and underestimation, respectively. MACE = major adverse cardiac events.

With respect to MACE, cross-validation yielded an AUC of 0.714 (95% CI 0.676-0.752) for CS and a better AUC of 0.746 (95%CI 0.709-0.784) for CCS. Both models exhibited excellent calibration. Similar results were observed for all-cause death, cardiac death, and myocardial infarction. For revascularization, calibration worsened with CCS as compared with CS. Figure 4(A–E) listed the cross-validation AUCs, intercepts, and slopes for CCS and CS, respectively, with respect to clinical outcomes.

As shown in Table 3, when reclassifying patients with MACEs from the risk category of CS into CCS (≤10%, 10% to ≤20%, 20% to ≤40% and >40%), 51/234(22%) patients with events were moved to higher risk categories (upward) and 28/234 (11%) to lower risk categories (downward), thus resulting in a net gain of 10%. In patients without events, 164 were moved downward and 80 were moved upward, thus resulting in a net gain of 11%. Consequently, the category-dependent NRI was 21% (*Z* = 4.78, *p* < 0.001). Following the same procedure, the category-dependent NRIs for all-cause death, cardiac death, myocardial infarction, and revascularization were 52% (*p* < 0.001), 48% (*p* < 0.001), 31% (*p* < 0.001), and −29% (*p* < 0.001), respectively (Table 4). We also calculated the category-free NRIs and IDI for all endpoints, indicating that CCS better predicted clinical outcomes than CS as shown in Table 4. With respect to all-cause death, cardiac death, myocardial infarction, and revascularization, reclassifications were observed (data not shown).

**Table 3.** MACE reclassification from CatLet score to Clinical CatLet score.

	Predicted risk on Clinical CatLet score				
Predicted risk on CatLet score	≤10%	10%∼	20%∼	40%∼	Total
Patients with MACEs					
≤10%	5	3^†^	0^†^	0^†^	8
10%∼	5^‡^	55	14^†^	1^†^	75
20%∼	0^‡^	10^‡^	41	33^†^	84
40%∼	0^‡^	1^‡^	12^‡^	54	67
Total	10	69	67	88	234
Patients without MACEs					
≤10%	69	15^§^	0§	0§	84
10%∼	74	334	38§	0§	446
20%∼	1	60^¶^	98	27§	186
40%∼	0	0^¶^	29^¶^	39	68
Total	144	409	165	66	784

Notes: Patients in the diagonal cells had the same risk category between both models. The gray shadow indicated that the predicted probability of the Clinical CatLet score was moving in the right direction: more patients with MACEs moved to higher risk while more patients without MACEs moved to lower risk. ^†^moved to higher risk, *n* = 51; ^‡^moved to lower risk, *n* = 28; NRI for MACE = 23/234 = 9.8%. ^¶^moved to lower risk, *n* = 164; ^§^moved to higher risk, *n* = 80; NRI for MACE free = 84/784 = 10.7%. NRI = net reclassification index. Other abbreviations as in previous tables.

**Table 4.** Improved performance of Clinical CatLet score as compared with CatLet score.

Outcomes	MACE	All-cause death	Cardiac death	Myocardial infarction	Revascularization
No. of participants	1018	1018	1018	1018	1018
No. of events	234	130	93	133	131
Category-dependent NRI	20.5% (Z = 4.78, *P* < 0.01)	51.6% (Z = 7.95, <0.01)	48.3% (Z = 6.43, *P* < 0.01)	31.0% (Z = 5.40, *P* < 0.01)	−28.8% (Z=−5.44, *P* < 0.01)
Non-event NRI	10.7%	13.1%	4.2%	9.2%	−25.0%
Event NRI	9.8%	38.5%	44.1%	21.8%	−3.8%
Category-free NRI	35.5% (*P* < 0.01)	76.2% (*P* < 0.001)	97.4% (*P* < 0.001)	64.5% (*P* < 0.001)	16.1% 0.0859
Non-event NRI	22.7%	40.8%	47.9%	27.7%	11.5%
Event NRI	12.8%	35.4%	50.5%	36.8%	4.6%
IDI	0.0577 (*P* < 0.01)	0.1741 (*P* < 0.01)	0.1910 (*P* < 0.01)	0.0809 (*P* < 0.01)	0.0082 0.0065

IDI = integrated discrimination index. Other abbreviations as in previous tables.

## Discussions

To our knowledge, this is the first study to validate the efficacy of CS in outcome predictions at four years of follow-up focused on AMI patients with symptom onset >12 h. The main findings of the current study demonstrated that CS and, to a greater extent, CCS were able to risk-stratify long-term outcomes in AMI patients presenting >12 h after symptom onset.

The current study had comparable inclusion and exclusion criteria to those of the first CatLet validation study. However, the mean CS in the current study was 16, lower than the 20 reported in the first CatLet validation study. This observation is not surprising considering the lower average of 1.70 ± 0.94 lesions/patient in the current study vs. 2.11 ± 1.21 in the first CatLet validation study [11].

This CatLet angiographic scoring system tends to comprehend the variable coronary anatomy, severity of a coronary flow-limiting lesion, and its subtended myocardial territory [10]. Our previous study revealed that CS independently predicted clinical outcomes in patients with AMI presenting within 12 h [11]. Therefore, it is not surprising that CS remains an independent predictor of clinical outcomes for AMI patients presenting >12 h. CCS is calculated by multiplying the anatomically-based CS by the clinically-based ACEF I score instead of the ACEF II score, as the ACEF II score had similar or poor performance in predicting clinical outcomes for CAD patients [26]. Previous studies have demonstrated that models incorporating both anatomical and clinical factors performed better in outcome predictions than either, such as the Clinical SYNTAX score [4], global risk classification [27], and New Risk Stratification (NERS) [28]. Therefore, it is anticipated that, to a greater extent, CCS independently predicted the clinical outcomes revealed in the current study. These findings are consistent with those of our previous study, in which age, serum creatinine, and left ventricular ejection fraction improved the performance of CS in terms of outcome predictions for AMI patients presenting within 12 h [29]. For harder events such as all-cause death and cardiac death, the improvement in discrimination and calibration was particularly prominent with CCS, indicating that these harder events were more affected by clinical variables. A previous study also showed that clinical variables substantially improved the predictive ability for 1-year all-cause mortality compared to the anatomical SYNTAX score in isolation [4]. Nevertheless, CCS had a diminished ability to explain the ischemia-driven revascularization compared with CS, which was consistent with a previous observation that the SYNTAX score, also an anatomically-based score, reflected repeat revascularization well, and that the Clinical SYNTAX score, however, failed to show any improvement [30]. Both CS and CCS did not substantially alter their predictive values of clinical outcomes after adjustment for a broad spectrum of risk factors.

Of note, both CS and CCS were well calibrated in the current study with respect to MACE, all-cause death, cardiac death, and myocardial infarction, with slopes close to 1 and intercepts close to 0. Regarding revascularization, however, CCS systematically overestimated its predictive value, with a slope of 0.841.

## Study limitations

As a retrospective study, the findings should be considered hypothesis-generating and unmeasured confounding factors are unavoidable although we have adjusted for a broad spectrum of risk factors. Another limitation is the selected population of patients with AMI presenting later than 12 h. Our first validation study focused on patients with AMI presenting within 12 h and demonstrated the utility of CS in outcome predictions [11]. The current study focused on a completely different CAD population, AMI patients presenting >12 h and undergoing PCI, and also validated its utility in outcome predictions. Therefore, the current study extends the application of CS to clinical practice. We will also separately investigate the values of CS in the prior MI and CTO populations in the near future. Lastly, we did not collect the mean time between symptom onset and PCI procedure, which would affect the association of CS with clinical outcomes. However, the current study is homogeneous in AMI patients arriving later than 12 h, which would minimize these effects.

## Conclusions

The utility of CS and CCS have been further validated with respect to 4-year outcome predictions in patients with AMI presenting >12 h after symptom onset. These findings have indicated that both anatomical and clinical variables should be considered in decision-makings on management of patients with AMI presenting later than 12 h after symptom onset.

## Acknowledgement

None.

## The author contribution statement

He YM and Xu JP conceived and designed this manuscript; He YM, Masuda S, Xu JP, and Sun BC collected the data; He YM, Jiang TB, Ge JB, and Sun BC analyzed the data; He YM drafted the manuscript and revised it critically; and all authors approved this paper to be published and agreed to be accountable for all aspects of this work.

## Authorship statement

The manuscript has been read and approved by all authors, the requirements for authorship have been met, and each author believes that the manuscript represents honest work.

## IRB information

Both studies complied with the Declaration of Helsinki regarding investigation in humans and was approved by the institute review board of Soochow University (reference numbers 2020048).

## Supplementary Material

Supplemental Material

## Data Availability

Data will be shared if requested appropriate.
