# Supplementary material for: CatLet score and clinical CatLet score as predictors of long-term outcomes in patients with acute myocardial infarction presenting later than 12 hours from symptom onset
Source: Ann Med. 2024 May 13;56(1):2349190. doi: 10.1080/07853890.2024.2349190 (PMC11095273; doi:10.1080/07853890.2024.2349190)
Supplement: Supplemental Material [file IANN_A_2349190_SM6933.docx]

**SUPPLEMENTAL MATERIALS**

**Article title:** CatLet score and Clinical CatLet score and their associations with long-term outcomes for patients with acute myocardial infarction presenting later than 12 hours from symptom onset

**Authors:** Yong-Ming He^1^, MD, PhD, FACC, FESC; Shinichiro Masuda MD^3^; Ting-Bo Jiang^1^, MD, PhD; Jun-Bo Ge^2^, MD, PhD.

**Contents:**

Supplementary figure S1

Supplementary figure S2

Supplementary figure S3

Supplementary figure S4

Supplementary table S1

The calculation of CatLet score is illustrated below. After definition of LAD, Dx, or RCA type, the calculator will automatically display the coronary segments and their weightings. The lesion and its adverse characteristics will be further evaluated ([www.catletscore.com](http://www.catletscore.com)).


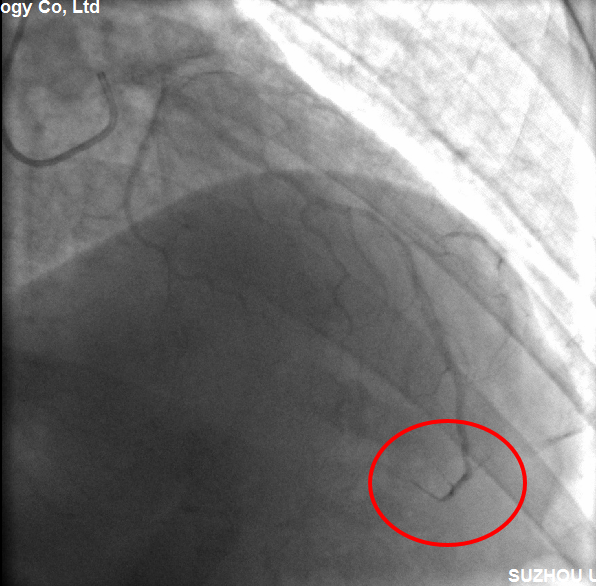


Figure S1. Left anterior descending artery (LAD) reaches the apex, and is adjudicated as average LAD.


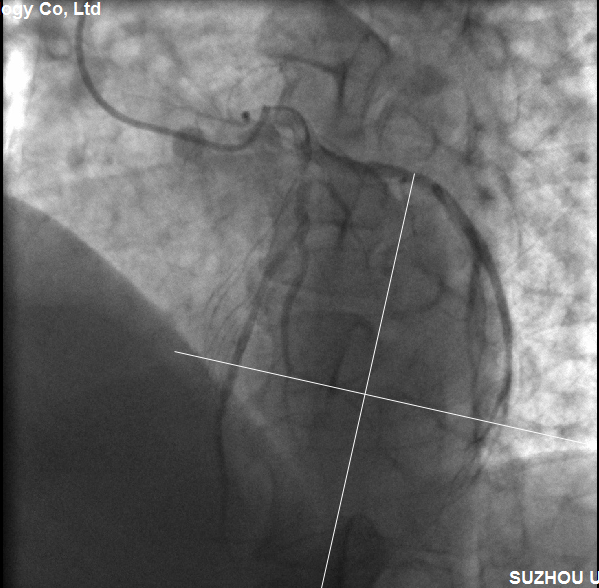


Figure S2. Diagonal branches (Dx) distribute over around three quadrants, and adjudicated as intermediate Dx.


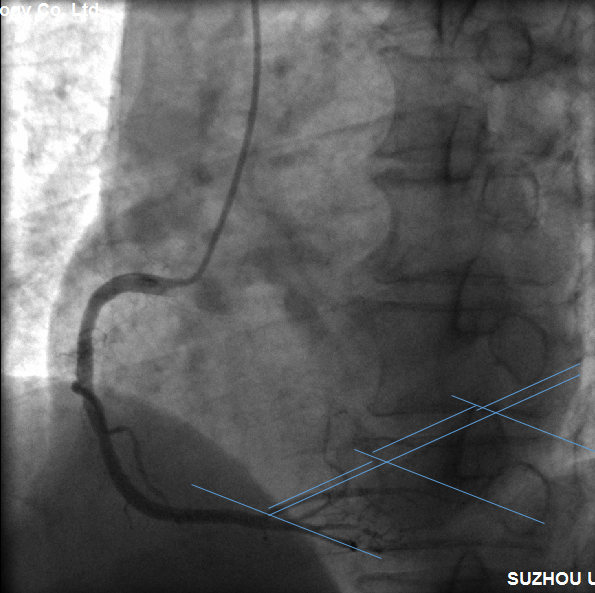


Figure S3. In the view of left anterior oblique 45, posterolateral vessels off right coronary artery (RCA) distribute over around the first third of left atrioventricular sulcus, and adjudicated as small RCA.


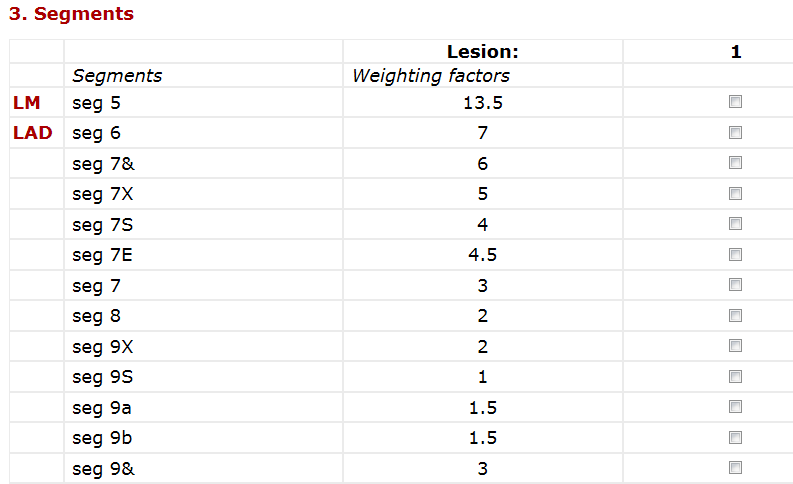


Figure S4. According to the algorithm of CatLet angiographic scoring system, the proximal lesion on the LAD (segment 6) is scored as 7.0×2=14 with an average LAD, intermediate Dx, and small RCA. The CatLet score calculator is available at [www.catletscore.com](http://www.catletscore.com).

Supplementary Table S1. Multivariable-adjusted hazard ratios (95%CI) for predictors with respect to outcomes.

| Outcomes | Predictors | HR(95%CI) | Z | P values |
| --- | --- | --- | --- | --- |
| MACE | CatLet score | 1.06(1.04-1.07) | 6.47 | <0.001 |
|  | Age, years | 1.02(1.01-1.04) | 3.06 | 0.002 |
|  | Creatinine, mg/dL | 1.19(1.02-1.38) | 2.23 | 0.026 |
|  | LVEF, % | 0.97(0.96-0.98) | -4.31 | <0.001 |
|  | Sex, male | 0.88(0.61-1.28) | -0.66 | 0.506 |
|  | Type 2 diabetes | 1.15(0.86-1.53) | 0.91 | 0.361 |
|  | Primary hypertension | 0.86(0.62-1.17) | -0.97 | 0.331 |
|  | Stroke | 1.17(0.74-1.85) | 0.67 | 0.504 |
|  | Smoking | 0.91(0.75-1.09) | -1.02 | 0.308 |
|  | Heavy calcification | 0.95(0.67-1.35) | -0.28 | 0.777 |
|  | Alcohol intake | 0.90(0.74-1.10) | -1.05 | 0.293 |
|  | Complete revascularization | 0.63(0.46-0.86) | -2.93 | 0.003 |
| All-cause death | CatLet score | 1.05(1.02-1.07) | 4.00 | <0.001 |
|  | Age, years | 1.07(1.05-1.10) | 6.35 | <0.001 |
|  | Creatinine, mg/dL | 1.46(1.27-1.67) | 5.46 | <0.001 |
|  | LVEF, % | 0.95(0.93-0.96) | -5.85 | <0.001 |
|  | Sex, male | 0.65(0.40-1.04) | -1.81 | 0.07 |
|  | Type 2 diabetes | 1.44(0.99-2.11) | 1.90 | 0.057 |
|  | Primary hypertension | 0.85(0.54-1.36) | -0.67 | 0.503 |
|  | Stroke | 1.57(0.90-2.71) | 1.60 | 0.11 |
|  | Smoking | 1.10(0.85-1.43) | 0.75 | 0.455 |
|  | Heavy calcification | 0.93(0.60-1.43) | -0.34 | 0.733 |
|  | Alcohol intake | 0.71(0.51-0.98) | -2.11 | 0.035 |
|  | Complete revascularization | 0.70(0.46-1.07) | -1.64 | 0.102 |
| Cardiac death | CatLet score | 1.07(1.04-1.09) | 5.06 | <0.001 |
|  | Age, years | 1.08(1.05-1.11) | 5.50 | <0.001 |
|  | Creatinine, mg/dL | 1.47(1.24-1.75) | 4.40 | <0.001 |
|  | LVEF, % | 0.93(0.91-0.95) | -6.29 | <0.001 |
|  | Sex, male | 0.47(0.27-0.82) | -2.66 | 0.008 |
|  | Type 2 diabetes | 1.29(0.82-2.04) | 1.11 | 0.269 |
|  | Primary hypertension | 0.59(0.35-1.00) | -1.96 | 0.05 |
|  | Stroke | 1.82(0.97-3.40) | 1.87 | 0.062 |
|  | Smoking | 1.13(0.83-1.54) | 0.75 | 0.451 |
|  | Heavy calcification | 0.77(0.46-1.28) | -1.01 | 0.312 |
|  | Alcohol intake | 0.77(0.52-1.14) | -1.29 | 0.197 |
|  | Complete revascularization | 0.68(0.41-1.13) | -1.48 | 0.138 |
| Myocardial infarction | CatLet score | 1.06(1.04-1.08) | 5.15 | <0.001 |
|  | Age, years | 1.04(1.02-1.06) | 4.02 | <0.001 |
|  | Creatinine, mg/dL | 1.38(1.18-1.61) | 4.12 | <0.001 |
|  | LVEF, % | 0.96(0.94-0.97) | -4.85 | <0.001 |
|  | Sex, male | 0.67(0.42-1.09) | -1.61 | 0.108 |
|  | Type 2 diabetes | 1.27(0.86-1.86) | 1.20 | 0.23 |
|  | Primary hypertension | 0.61(0.41-0.92) | -2.35 | 0.019 |
|  | Stroke | 1.51(0.87-2.62) | 1.47 | 0.142 |
|  | Smoking | 0.99(0.77-1.27) | -0.09 | 0.926 |
|  | Heavy calcification | 0.72(0.45-1.16) | -1.36 | 0.175 |
|  | Alcohol intake | 0.94(0.72-1.22) | -0.47 | 0.636 |
|  | Complete revascularization | 0.72(0.48-1.08) | -1.60 | 0.11 |
| Revascularization | CatLet score | 1.05(1.03-1.08) | 4.22 | <0.001 |
|  | Age, years | 1.00(0.98-1.01) | -0.59 | 0.554 |
|  | Creatinine, mg/dL | 0.91(0.60-1.36) | -0.48 | 0.631 |
|  | LVEF, % | 1.00(0.98-1.02) | 0.00 | 0.997 |
|  | Sex, male | 1.53(0.89-2.63) | 1.52 | 0.128 |
|  | Type 2 diabetes | 1.08(0.72-1.62) | 0.38 | 0.701 |
|  | Primary hypertension | 1.10(0.73-1.65) | 0.45 | 0.656 |
|  | Stroke | 0.84(0.40-1.74) | -0.47 | 0.637 |
|  | Smoking | 0.83(0.66-1.06) | -1.49 | 0.137 |
|  | Heavy calcification | 1.23(0.74-2.03) | 0.81 | 0.419 |
|  | Alcohol intake | 0.93(0.74-1.18) | -0.57 | 0.568 |
|  | Complete revascularization | 0.61(0.41-0.92) | -2.36 | 0.018 |

Notes: CatLet= Coronary Artery Tree description and Lesion Evaluation and Treatment System; MACE=major adverse cardiac events; and LVEF= left ventricular ejection fraction. For continuous variables, the HRs were calculated according to per 1SD increase.
